# Direct Neuronal Glucose Uptake Is Required for Contextual Fear Acquisition in the Dorsal Hippocampus

**DOI:** 10.3389/fnmol.2017.00388

**Published:** 2017-11-21

**Authors:** Liang Kong, Yan Zhao, Wen-Juan Zhou, Hui Yu, Shuai-Wen Teng, Qi Guo, Zheyu Chen, Yue Wang

**Affiliations:** ^1^Department of Cell and Neurobiology, Shandong Provincial Key Laboratory of Mental Disorders, School of Basic Medical Science, Shandong University, Jinan, China; ^2^Department of Clinical Laboratory, Affiliated Hospital of Shandong University of Traditional Chinese Medicine, Jinan, China

**Keywords:** glucose metabolism, ANLS, contextual fear conditioning, GLUT3, DH

## Abstract

The metabolism of glucose is a nearly exclusive source of energy for maintaining neuronal survival, synaptic transmission and information processing in the brain. Two glucose metabolism pathways have been reported, direct neuronal glucose uptake and the astrocyte-neuron lactate shuttle (ANLS), which can be involved in these functions simultaneously or separately. Although ANLS in the dorsal hippocampus (DH) has been proved to be required for memory consolidation, the specific metabolic pathway involved during memory acquisition remains unclear. The DH and amygdala are two key brain regions for acquisition of contextual fear conditioning (CFC). In 2-NBDG experiments, we observed that 2-NBDG-positive neurons were significantly increased during the acquisition of CFC in the DH. However, in the amygdala and cerebellum, 2-NBDG-positive neurons were not changed during CFC training. Strikingly, microinjection of a glucose transporter (GLUT) inhibitor into the DH decreased freezing values during CFC training and 1 h later, while injection of a monocarboxylate transporter (MCT) inhibitor into the amygdala also reduced freezing values. Therefore, we demonstrated that direct neuronal glucose uptake was the primary means of energy supply in the DH, while ANLS might supply energy in the amygdala during acquisition. Furthermore, knockdown of GLUT3 by a lentivirus in the DH impaired the acquisition of CFC. Taken together, the results indicated that there were two different glucose metabolism pathways in the DH and amygdala during acquisition of contextual fear memory and that direct neuronal glucose uptake in the DH may be regulated by GLUT3.

## Introduction

It is well known that a highly active brain accounts for at least 20% of the body’s daily energy consumption. In the central nervous system, the nearly exclusive source of energy is glucose aerobic metabolism. Most of this energy is utilized to sustain synaptic transmission, neuronal survival and information processing underlying multiple behaviors such as learning and memory, emotion and sensorimotor activity (Benarroch, 2014). The precise connection of neurons, astrocytes and endothelial cells plays a crucial role in coupling energy supply with neuronal activity (Magistretti and Allaman, 2015). To maintain these high energy demands, neurons can take up glucose directly; alternatively, astrocytes can convert glucose into lactate and supply it to neurons (Suzuki et al., 2011; Lundgaard et al., 2015). However, the exact methods of energy supply during different stages of neuronal function and in different brain areas remain unclear (DiNuzzo et al., 2010; Bélanger et al., 2011; Mangia et al., 2011).

It has been almost 20 years since the astrocyte-to-neuron lactate shuttle (ANLS) was proposed as a means of sustaining neuronal activity. Multiple lines of evidence show that lactate is able to sustain neuronal activity in cultured neurons and in anesthetized animals (Schurr et al., 1988; Pellerin and Magistretti, 1994; Wyss et al., 2011). Glucose transporter 1 (GLUT1), monocarboxylate transporter 1 (MCT1) and MCT4 are highly enriched in astrocytes. In the central nervous system, glycogen, which is only found in astrocytes, can be rapidly converted to lactate and transported through the extracellular medium by MCT1 and MCT4; lactate can then be absorbed by neurons as fuel via MCT2 (Suzuki et al., 2011). Although ANLS is a reasonable and efficient model for glucose metabolism in the brain, there was never lack of doubt (Chih et al., 2001; Patel et al., 2014). For example, due to the concentrations and kinetic characteristics of GLUTs and MCTs, lactate derived from astrocytes may not be transported into neurons under physiological conditions (Mangia et al., 2009, 2011). GLUT3 is specially expressed in neurons for direct glucose uptake *in situ*. Recent studies using two-photon imaging of a near-infrared 2-deoxyglucose analog (2DG-IR) reveal that glucose is taken up preferentially by neurons in awake mice and that whisker stimulation significantly increases glucose in neurons but not astrocytes (Lundgaard et al., 2015). Hexokinase, a key metabolic enzyme in glycolysis, is more highly expressed in neurons than in astrocytes.

Learning and memory is a sequential process that has several stages, including acquisition, consolidation and retrieval (Kandel et al., 2014). Distinct molecular mechanisms have been reported in different stages (Abel and Lattal, 2001; Johansen et al., 2011). For example, the acquisition stage has been shown to be related to post-translational modifications, and the consolidation of long-term memory (LTM) relies on protein synthesis (Hernandez and Abel, 2008; Maren et al., 2013). It has been recognized that GLUT1 is critical in the consolidation stage of memory in the bead discrimination paradigm (Gibbs et al., 2008). Meanwhile, in inhibitory avoidance experiments using MCT interference, ANLS has been shown to be required for consolidation in the dorsal hippocampus (DH; Suzuki et al., 2011). However, the exact metabolic pathway involved during memory acquisition in related brain regions is unknown.

In this study, contextual fear conditioning (CFC) was applied as a classic associative memory model. The energy metabolism pathways in two crucial areas, the DH and amygdala for contextual fear memory, were investigated. We found that direct neuronal glucose uptake was the primary energy supply method in the DH, while ANLS was involved in the amygdala during memory acquisition. In addition, unlike metabolic pathways in memory consolidation, GLUT3-directed neuronal glucose uptake was necessary for CFC acquisition in the DH.

## Materials and Methods

### Animals

Male C57BL/6 mice, consistently 8 weeks old and weighting 25–30 g, were used in the following experiments. Animals were maintained at 22°C under 12:12 light/dark cycles with enough food and water. All procedures were in accordance with the National Institutes of Health *Guide for the Care and Use of Laboratory Animals* and were approved by the Institutional Animal Care and Use Committees of Shandong University.

### Surgery and Microinjection

2-NBDG (N13195) was purchased from Invitrogen and dissolved in sterile normal saline. Other chemicals were from Sigma-Aldrich Inc., including cytochalasin B (C6762) and 4-CIN (C2020). These two drugs were dissolved in DMSO and diluted with sterile normal saline. A third-generation lentiviral vector system used in the experiments includes a transfer vector PGW and three package plasmids pMDL/pRRE, VSV-G and pRSV-REV. RNA interference plasmids used to package lentivirus were purchased from Thermo Scientific Open Biosystems. The GLUT3 shRNA forward sequences 5′-GCA GGC GTG GTC AAT ACT ATT CAA GAG ATA GTA TTG ACC ACG CCT GCT TTT TT-3′ and reverse sequences 5′-AAT TAA AAA AGC AGG CGT GGT CAA TAC TAT CTC TTG AAT AGT ATT GAC CAC GCC TGC GGC C-3′ were annealed and ligated into pSilencer 1.0 vector at the ApaI and EcoRI site. Lenti-siGLUT3 was then constructed by inserting the U6 promoter and the GLUT3 shRNA sequences derived from pSilencer 1.0 into the PGW lentiviral vector. The PGW lentiviral vector also contains a GFP reporter gene. High titer lentiviruses were generated by transfection of 293T with the above lentiviral vectors.

To perform microinjection, the 8-week-old mice were first anesthetized with 5% chloral hydrate. Then, cannulas (26-gauge guide) were implanted in the bilateral DH, amygdala or cerebellum. Stereotaxic coordinates were as follows: the DH (AP −1.75 mm, lateral ±1.35 mm, and dorsoventral −2.25 mm), the basolateral amygdala (AP −1.4 mm, L ±3.25 mm, DV −5.05 mm) and the cerebellum (AP −6.5 mm, L 0 mm, DV −2.0 mm). Mice were kept in cage for a recovery period of 1 week before microinjection and behavior test. At the indicated time points before fear training, infusion needles connected with a microinjection pump (KDS200, KD Scientific) were used to inject drugs through the cannula into the designated position. The needle was left in the cannula for two additional minutes after injection to ensure complete diffusion of drugs or lentivirus. The brain volume with dye spread almost equal in our experimental conditions, which every brain region received equal volume of drugs at the same location for the same diffusion time. We used the following concentrations: cytochalasin B (dissolved in 0.1% dimethyl sulfoxide, 0.5 mM, 0.5 μL/side), 4-CIN (dissolved in 0.1% dimethyl sulfoxide, 60 μM, 0.5 μL/side), 2-NBDG (dissolved in sterile saline, 10 mM, 0.5 μL/side) and lentivirus (Lenti-siSCR or Lenti-siGLUT3, 10^9^ TU/mL, 1 μL/side).

### Contextual Fear Conditioning

Fifteen minutes after microinjection of inhibitors of GLUT and MCT, mice were subjected to CFC as previously described (Xu et al., 2015). Besides, in microinjection of lentivirus experiments, mice were 8 weeks old during surgery and microinjection. To ensure that lentivirus was fully expressed, mice were kept in cage for another 4 weeks before behavior test. In brief, mice were put into a test chamber (25 × 25 × 25 cm, Panlab) and habituated to the environment for 2 min without stimulation (habituation). Mice were then subjected to three 0.7 mA, 2 s foot shocks through a stainless steel grid floor. Consecutive foot shocks were separated by a 1 min interval. Mice were returned to their home cage 1 min after the final shock. The foot shock is regarded as the US stimuli and is generated by a programmable animal shocker, and the chamber context is the CS stimuli. Short-term memory (STM) and LTM were tested 1 and 24 h after fear training, respectively. Fear memory was evaluated by measuring mice freezing value. To delineate mice acquisition curve in each group, the freezing value in habituation stage (baseline) and three intervals after each foot shock was also measured.

### Open Field Test

Locomotor activity was assessed in the open field test. The apparatus consisted of 40 cm × 40 cm black box and a camera at the top. Mice were placed in the center of the field and a videotracking system (Smart) was used to score the distance traveled of each animal for 10 min. The locomotor activity was evaluated by measuring mice total moving distance in the area.

### Tissue Preparation and Western Blotting

Mice brains were rapidly removed after euthanasia. Both the DH and amygdala were dissected immediately on ice. Samples were then homogenized in ice-cold lysis buffer containing 150 mM NaCl, 10 mM Tris-HCl (pH 7.6), 1 mM EDTA, 1% NP-40, proteases and phosphatase inhibitors. The extracts were centrifuged at 16,000 *g* at 4°C for 20 min, and the supernatants were collected. Sample protein quantification was performed using the bicinchoninic acid (BCA) assay (Pierce Biotechnology). Total protein of each sample was normalized and 20 μg protein was loaded to each well.

Primary antibodies were used at the following dilutions: rabbit anti-GLUT3 (1:500, GT31-A, Alpha Diagnostic International); rabbit anti-β-actin (1:1000, Cell Signaling Technology). Horseradish peroxidase (HRP)-conjugated goat anti-rabbit secondary antibodies were purchased from Calbiochem. Blots were visualized using an ECL chemiluminescence system (Millipore). Finally, Quantity One software was used to analyze the densitometry of the bands.

### Immunohistochemistry Staining

After anesthetization with 5% chloral hydrate, mice were perfused with 0.9% saline and 4% paraformaldehyde. Brains were removed and infiltrated with 30% sucrose. Brains were subsequently frozen and 40 μm serial coronal sections were cut throughout the DH, amygdala and cerebellum using a freezing microtome. Coronal slices were blocked in 5% donkey serum solution containing 0.3% Triton X-100 and incubated with different primary antibodies overnight at 4°C. Primary antibodies were used at the following dilutions: mouse anti-NeuN (1:1000, Millipore), rabbit anti-GFAP (1:1000, Millipore), rabbit anti-GFP (1:1000, Invitrogen). Slices were then incubated with donkey anti-rabbit Alexa 488, donkey anti-rabbit Alexa 594 or donkey anti-mouse Alexa 594 for 1 h at room temperature. Images were captured using fluorescence microscopy (Nikon 80i) and imaging computer program (NIS-Elements BR, Nikon). In 2-NBDG experiments, one section was taken every third slice (Yu et al., 2009; Wang et al., 2014). According to the atlas of mice brain, total six sections were selected from the DH, amygdala and cerebellum, respectively (DH, −1.25 mm to −2.25 mm; amygdala, −0.9 mm to −1.9 mm; cerebellum, −6.0 mm to −7.0 mm from the Bregma). Four areas were randomly captured per section under the 20× objective. 2-NBDG^+^NeuN^+^ and 2-NBDG^+^/GFAP^+^ cells were counted in each area and mean value was calculated from these areas.

### Statistical Analysis

SPSS statistical program (version 19.0) software was used for data analysis. The statistical methods used in the experiments included Student’s *t* test, one-way and two-way ANOVAs, followed by LSD or Dunnett’s T3 *post hoc* test to compare differences between multiple groups spontaneously. Data are presented as means ± SEM and the significance threshold was set at *p* < 0.05.

## Results

### 2-NBDG Experiments Revealed That Direct Neuronal Uptake of Glucose May Be the Primary Energy Source in the DH during CFC Acquisition

**Figure 1:**
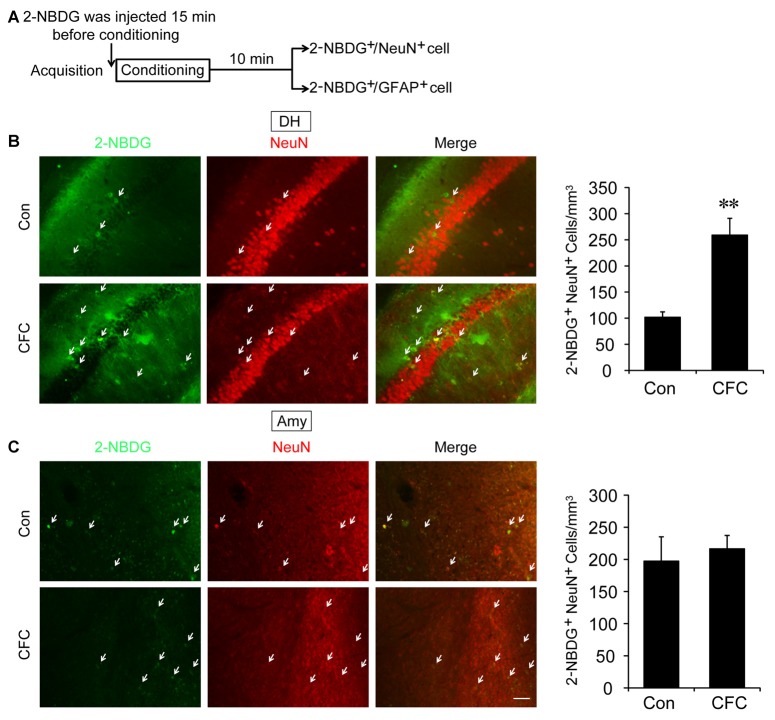
The destination of glucose under basal condition and contextual fear conditioning (CFC) training in the dorsal hippocampus (DH) and amygdala. **(A)** Schematic representation of the experiments to investigate the destination of glucose during CFC training in both the DH and amygdala. **(B,C)** Representative fluorescence images and relative quantification of 2-NBDG^+^/NeuN^+^ positive cells after microinjection of 2-NBDG into the DH or amygdala and CFC training (*n* = 4 per group; errors represent SEM, **p < 0.01 vs. control group). White arrows represent 2-NBDG^+^/NeuN^+^ cells. Scale bar = 50 μm.

**Figure 2:**
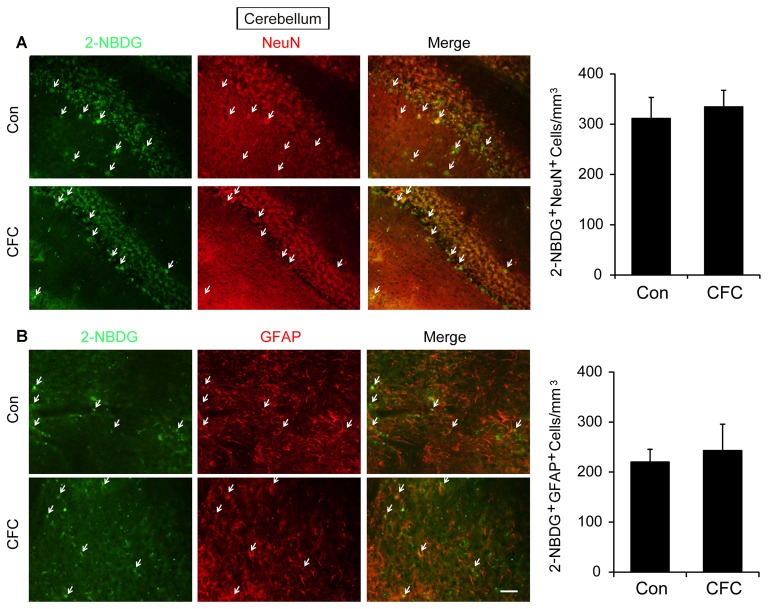
The destination of glucose under basal condition and CFC training in the cerebellum. **(A)** Representative fluorescence images and relative quantification of 2-NBDG^+^/NeuN^+^ positive cells after microinjection of 2-NBDG into the cerebellum and CFC training (*n* = 4 per group; errors represent SEM). White arrows represent 2-NBDG^+^/NeuN^+^ cells. **(B)** Representative fluorescence images of 2-NBDG^+^/GFAP^+^ positive cells after microinjection of 2-NBDG into the cerebellum and CFC training. White arrows represent 2-NBDG^+^/GFAP^+^ cells. Scale bar = 50 μm.

The DH and amygdala are two crucial regions of contextual fear circuit (Phillips and LeDoux, 1992; Maren et al., 2013). Functional magnetic resonance imaging (fMRI) studies have reported that the DH and amygdala are highly active during acquisition of fear conditioning (Alvarez et al., 2008; Marschner et al., 2008; Lang et al., 2009). This suggested that both of them may demand high levels of metabolic activity. Glucose aerobic metabolism has been regarded as the only source of energy. The fluorescent glucose-tracer 2-NBDG can be transported into both neurons and astrocytes (Itoh et al., 2004; Ferreira et al., 2011; Jakoby et al., 2014). To visually investigate the destination of glucose absorbed during fear training in CFC acquisition-related (DH and amygdala) and -unrelated brain regions (cerebellum), 2-NBDG was used in the following experiments (Wang et al., 2014). Fifteen minutes before fear condition training, 2-NBDG was injected into the DH, amygdala and cerebellum, and 10 min after training, the 2-NBDG-positive cells were measured using fluorescence microscopy (Figure 1A). We found that during the basal stage (control group) and CFC training, 2-NBDG-positive cells in the DH were nearly completely merged with NeuN-positive cells (Figure 1B) and rarely overlapped with GFAP-positive cells (Supplementary Figure S1A), suggesting that 2-NBDG tends to be absorbed by neurons in the DH. In addition, CFC training significantly increased 2-NBDG-positive neurons in the DH (*p* < 0.01; Figure 1B). In the amygdala, 2-NBDG was similarly nearly completely absorbed into neurons during the basal stage (Supplementary Figure S1B). However, there was no difference in the number of 2-NBDG-positive neurons between naïve (control group) and CFC training mice (*p* = 0.674; Figure 1C). In the cerebellum, the number of 2-NBDG-positive neurons was likewise unchanged after CFC training (*p* = 0.69; Figure 2A). Although a certain number of 2-NBDG-positive astrocytes was observed, CFC training did not change the number of 2-NBDG-positive astrocytes in the cerebellum (*p* = 0.717; Figure 2B). Therefore, our results demonstrated that neurons specific in the DH may absorb more glucose during associative learning acquisition. However, due to the controversial nature of 2-NBDG uptake rate in different cell types (Itoh et al., 2004; Barros et al., 2009a,b; Jakoby et al., 2014; Lundgaard et al., 2015), more experiments were carried out to further verify this finding.

### Direct Neuronal Glucose Uptake Was Required for CFC Acquisition in the DH While ANLS Was Involved in Acquisition in the Amygdala

**Figure 3:**
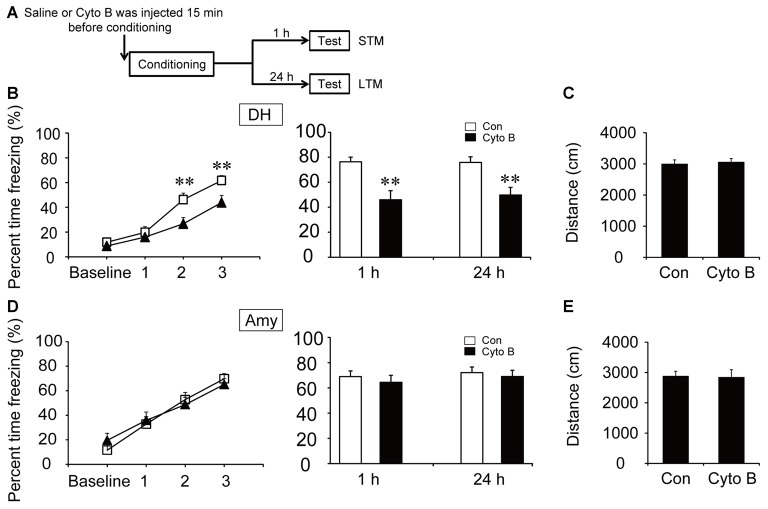
Glucose transporter (GLUT) inhibitor impaired the acquisition of CFC in the DH. **(A)** Schematic representation of the experimental schedule to investigate the effect of GLUTs on CFC memory acquisition in both the DH and amygdala, respectively. Mice received bilateral injections of the GLUT inhibitor cytochalasin B or saline 15 min before CFC training. **(B,D)** CFC acquisition curve, short-term memory (STM) and long-term memory (LTM) were evaluated after microinjection of cytochalasin B or saline into the DH or amygdala (*n* = 13–14 per group; errors represent SEM, **p < 0.01 vs. control group). Baseline represents initial 2 min habituation stage, the three numbers (1, 2, 3) represent three intervals after each foot shock, respectively. **(C,E)** Locomotor activity was quantified after microinjection of cytochalasin B or saline into the DH or amygdala on open field test (*n* = 13–14 per group; errors represent SEM). Locomotor activity was evaluated by measuring mice total moving distance in 10 min.

**Figure 4:**
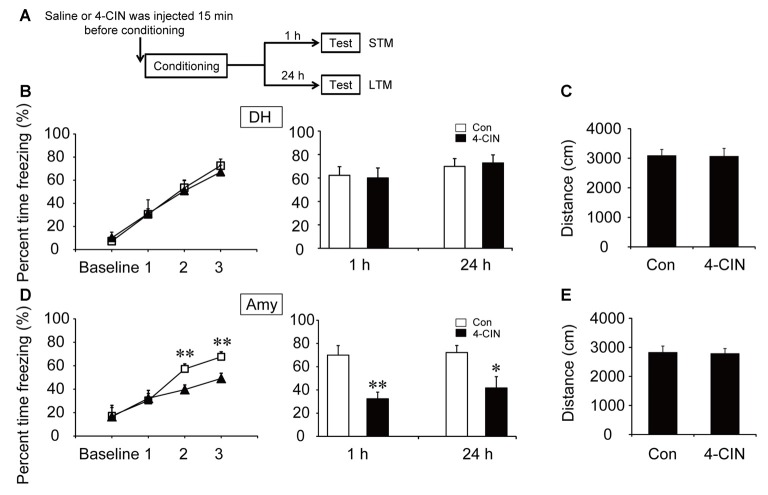
Monocarboxylate transporter (MCT) inhibitor impaired the acquisition of CFC in the amygdala. **(A)** Schematic representation of the experimental schedule for investigating the effect of MCTs on CFC memory acquisition in both the DH and amygdala, respectively. Mice received bilateral injections of the MCT inhibitor 4-CIN or saline 15 min before CFC training. **(B,D)** CFC acquisition curve, STM and LTM were evaluated after the microinjection of 4-CIN or saline into the DH or amygdala (*n* = 7–11 per group; errors represent SEM, *p < 0.05, **p < 0.01 vs. control group). Baseline represents initial 2 min habituation stage, the three numbers (1, 2, 3) represent three intervals after each foot shock, respectively. **(C,E)** Locomotor activity was quantified after microinjection of 4-CIN or saline into the DH or amygdala on open field test (*n* = 7–11 per group; errors represent SEM).

Direct neuronal glucose uptake and ANLS are two important energy supply pathways in the brain. To investigate the source of energy supply during CFC acquisition, STM and LTM were evaluated after injection of inhibitors of GLUT and MCT in the specific brain regions 15 min before contextual fear training (Figures 3A, 4A). The acquisition curve was depressed relative to the vehicle group after microinjection of GLUT antagonist (cytochalasin B) into the DH (*p* < 0.01). Moreover, freezing values on both STM and LTM tests significantly decreased after cytochalasin B injection in the DH (STM, *p* < 0.01; LTM, *p* < 0.01; Figure 3B). However, microinjection of cytochalasin B into the amygdala had no effect on the acquisition curve or freezing values on STM and LTM tests (Figure 3D; all *p* > 0.05). On the open field test, results indicated no reliable difference of moving distance between the vehicle group and the cytochalasin B-injected group (all *p* > 0.05), suggesting that the deficits in the above behaviors were not related to mouse locomotor activity (Figures 3C,E). These results suggested that glucose transport in the DH but not in the amygdala was necessary for fear memory acquisition.

To investigate the effect of ANLS on fear memory acquisition, the MCT inhibitor 4-CIN was used in the following experiments. It is noteworthy that the acquisition curve and freezing values of STM and LTM were not significantly different after microinjection of 4-CIN in the DH before contextual fear training (all *p* > 0.05; Figure 4B). However, the acquisition curve was impaired after the injection of MCT inhibitor in the amygdala (*p* < 0.01; Figure 4D). Injection of the MCT inhibitor also significantly decreased freezing values of STM and LTM in the amygdala after training (STM, *p* < 0.01; LTM, *p* = 0.013). In addition, microinjection of 4-CIN had no effect on mouse locomotor activity (all *p* > 0.05; Figures 4C,E). These results suggested that ANLS was necessary for fear memory acquisition in the amygdala but not in the DH during fear training. Furthermore, in the DH, the finding that suppression of ANLS had no effect on fear memory acquisition further demonstrated that direct neuronal glucose transport was necessary for CFC acquisition in the DH. Together, these findings demonstrated that energy supply pathways during fear memory acquisition were different in related brain regions.

### GLUT3 Is Involved in Neuronal Glucose Uptake and Fear Memory Acquisition in the DH

**Figure 5:**
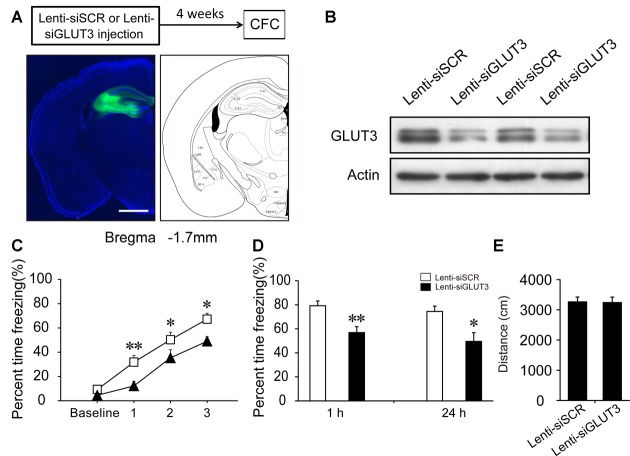
Neuronal GLUT3 drives contextual fear acquisition in the DH. **(A)** Schematic representation of the location and diffusion range of Lenti-siGLUT3 microinjected into the DH. GFP antibody was used to perform the immunofluorescence staining of GFP protein. Scale bar = 1 mm. **(B)** Representative immunoblots of GLUT3 protein levels in the DH after Lenti-siSCR or Lenti-siGLUT3 injection for 4 weeks. **(C,D)** CFC acquisition curve, STM and LTM were evaluated after microinjection of Lenti-siSCR or Lenti-siGLUT3 into the DH for 4 weeks (*n* = 8 per group; errors represent SEM, *p < 0.05, **p < 0.01 vs. control group). Baseline represents initial 2 min habituation stage, the three numbers (1, 2, 3) represent three intervals after each foot shock, respectively. **(E)** Locomotor activity was quantified 4 weeks after microinjection of lentivirus on open field test (*n* = 8 per group; errors represent SEM).

GLUT3 is mainly expressed in neurons. *In vitro* experiments have revealed that surface GLUT3 is rapidly increased after treatment with bicuculline (an inhibitor of GABA_A_ receptor) plus 4-AP (a voltage-gated potassium channel blocker), by approximately 20-fold within 30 min in cultured neurons (Ferreira et al., 2011). To investigate whether GLUT3-mediated neuronal glucose uptake participates in CFC acquisition, we generated a lentivirus expressing siRNA sequences against GLUT3 (Lenti-siGLUT3) and a control lentivirus with a scrambled sequence (Lenti-siSCR). To investigate the role of neuronal GLUT3 on fear memory acquisition, lentivirus was injected into the DH 4 weeks before fear condition training. First, the interference efficiency of Lenti-siGLUT3 was measured. In our experiments, GLUT3 decreased 50% 4 weeks after injection of Lenti-siGLUT3 (Figures 5A,B; Supplementary Figure S2). Mice injected with lentivirus subsequently underwent CFC training. Our results showed that microinjection of Lenti-siGLUT3 impaired acquisition curve compared with Lenti-siSCR-treated mice during CFC training in the DH (*p* < 0.05). Additionally, knockdown of GLUT3 significantly decreased freezing values of STM and LTM, compared with the control group (STM, *p* < 0.01; LTM, *p* = 0.011; Figures 5C,D). Besides, knockdown of GLUT3 has no effect on mouse locomotor activity (Figure 5E). Therefore, our results proved that GLUT3 directly dominated neuronal glucose uptake and fear memory acquisition in the DH.

## Discussion

Our results provide two new insights into the effect of glucose metabolic pathway on learning and memory. First, we found that neuronal direct uptake of glucose was the primary pathway in the DH, while ANLS might be involved in the amygdala during acquisition of CFC. Second, GLUT3 were involved in neuronal glucose uptake and contextual fear acquisition in the DH.

The brain primarily relies on glucose metabolism for energy supply to maintain neuronal function (Benarroch, 2014). Without sufficient energy supply, brain functions such as emotion, sensation, motor control and memory will be largely compromised (Mergenthaler et al., 2013). Glucose metabolism deficiencies can even accelerate neuropathological changes such as AD pathogenesis, neuroglycopenia and cognitive impairment (Cunnane et al., 2011; Glenn et al., 2014; Liu et al., 2015). The majority of researchers focus on two major glucose metabolic pathways: neuronal direct uptake of glucose and ANLS (Bélanger et al., 2011; Hall et al., 2012; Jakoby et al., 2014; Patel et al., 2014; Yang et al., 2014; Lundgaard et al., 2015). Heterogeneity in glucose metabolism pathways may exist in different brain areas and in different stages of higher brain function.

The DH and amygdala are regarded as two mainstays for CFC memory circuits (Maren et al., 2013), and may demand much energy supply during contextual fear training (Alvarez et al., 2008; Lang et al., 2009). Our results showed that in the DH but not the amygdala, CFC training significantly increased the number of 2-NBDG-positive neurons. Although the 2-NBDG uptake rate in different cell types is still controversial, it has been used to evaluate glucose transport in both neurons and astrocytes (Itoh et al., 2004; Jakoby et al., 2014). Several fluorescent glucose analog experiments have proved that glucose is preferentially taken up by astrocytes compared with neurons (Barros et al., 2009a,b; Jakoby et al., 2014). However, others believe that neurons absorb the majority of glucose (Itoh et al., 2004; Lundgaard et al., 2015). These discrepancies may result from distinct metabolic characteristics as well as different concentrations and incubation times of analogs. Consistent with the latter theory, our results suggested that neurons might prefer to absorb much more glucose than astrocytes to supply energy for memory acquisition in the DH. In addition, 30 min after microinjection of 2-NBDG, compared with overlap of 2-NBDG and GFAP in cerebellum, there were few 2-NBDG were found in astrocyte in both the DH and amygdala. This suggested that astrocyte may have a lower glucose uptake rate than neuron in the DH and amygdala. The unequal distribution of glucose in different cells in the DH and amygdala may ensure neuronal high activity to perform learning and memory, and emotion behaviors.

To further verify our conclusion, we used inhibitors of GLUT and MCT in our experiments. GLUTs and MCTs are two typical energy-related transporters (Simpson et al., 2007). GLUT3 has been regarded as the main transporter in the neuronal direct uptake of glucose. Alternatively, the ANLS hypothesis proposes that lactate can be transported outside of astrocytes via MCT1 and MCT4, and transported into neurons by MCT2 (Suzuki et al., 2011). We found that blocking lactate transport by injection of MCT inhibitor in the amygdala but not the DH impaired fear memory acquisition. Our results indicated that ANLS is the primary metabolic pathway in the amygdala but not the DH during CFC acquisition. In the meantime, inhibition of glucose transport in the DH but not the amygdala decreased fear acquisition. Altogether, these findings indicate that neuronal direct uptake of glucose in the DH is necessary for fear memory acquisition. In the amygdala, lactate derived from ANLS pathway may be crucial for fear memory acquisition, while the transport of glucose into neurons seems to have only a negligible effect. Certainly, given that the ANLS pathway remains controversial, we could not rule out the effects of other MCT-regulated pathways during contextual fear training in this study. In contextual fear circuits, the DH is the region for spatial information processing, while the amygdala mainly controls sensory inputs and fear formation (Ehrlich et al., 2009; Ciocchi et al., 2010; Redondo et al., 2014). Different functional components may depend on different metabolism pathways.

Additionally, in contrast to metabolic pathways involved in the consolidation stage, we demonstrated that GLUT3-mediated neuronal uptake of glucose is involved in CFC acquisition in the DH. Pierre J. Magistretti’s team has shown that ANLS is required for LTM by influencing consolidation in mouse inhibitory avoidance models (Suzuki et al., 2011). Lactate may be a potential substitute for glucose (Schurr et al., 1988; Wyss et al., 2011). The transport of lactate into neurons stimulates the expression of synaptic plasticity-related genes such as Arc, c-Fos and Zif268, which may contribute to regulation of consolidation (Yang et al., 2014). GLUT1, which is enriched in astrocyte and endothelial cells, is critical for memory consolidation in the bead discrimination paradigm in chickens (Gibbs et al., 2008). GLUT3 has a higher affinity for glucose than GLUT1 (Uldry and Thorens, 2004; Simpson et al., 2007). However, there is no direct evidence to elaborate the role of GLUT3 in memory acquisition. We found that knockdown of GLUT3 impaired fear acquisition in the DH. Previous *in vitro* experiments demonstrated that surface GLUT3 was rapidly increased after stimulation with bicuculline plus 4-AP, by approximately 20-fold within 30 min in cultured neuron (Ferreira et al., 2011). Consistent with this finding, we proved that GLUT3 may be required for fear acquisition in the DH. Compared with consolidation stage, transitory acquisition may require more energy to accomplish synaptic transmission. The high affinity for glucose of GLUT3 enables neurons to absorb sufficient glucose via glycolysis and supply abundant energy. Thus, in the DH, the acquisition and consolidation stages might be characterized by distinct metabolic pathways for sustaining energy supply. Artificial regulation of surface GLUT3 will be helpful for memory improvement.

In short, we concluded that the DH and amygdala depended on different glucose metabolism pathways during CFC acquisition. The heterogeneity of metabolic pathways may guarantee that the brain quickly and efficiently uses all energy sources to solve the emergency of energy exhaustion. In the future, more explicit characterization of these mechanisms will help us to develop effective therapeutic targets for the treatment of brain metabolic and memory disorders.

## Author Contributions

This work was mainly finished by LK. The other authors also have made significant contributions to this article.

## Conflict of Interest Statement

The authors declare that the research was conducted in the absence of any commercial or financial relationships that could be construed as a potential conflict of interest.

## Supplementary Material

FIGURE S1 There is no overlap of 2-NBDG^+^ and GFAP^+^ positive cells under basal condition and CFC training in the DH and amygdala. **(A)** Representative fluorescence images of 2-NBDG and GFAP after microinjection of 2-NBDG into the DH (*n* = 4 per group). **(B)** Representative fluorescence images of 2-NBDG and GFAP after microinjection of 2-NBDG into the amygdala (*n* = 4 per group). White arrows represent only 2-NBDG^+^ cells. Scale bar = 50 μm.

FIGURE S2 The original western blot images of Figure 5B.
