# Supplementary figures and images for: Direct Neuronal Glucose Uptake Is Required for Contextual Fear Acquisition in the Dorsal Hippocampus
Source: Front Mol Neurosci. 2017 Nov 21;10:388. doi: 10.3389/fnmol.2017.00388 (PMC5702440; doi:10.3389/fnmol.2017.00388)

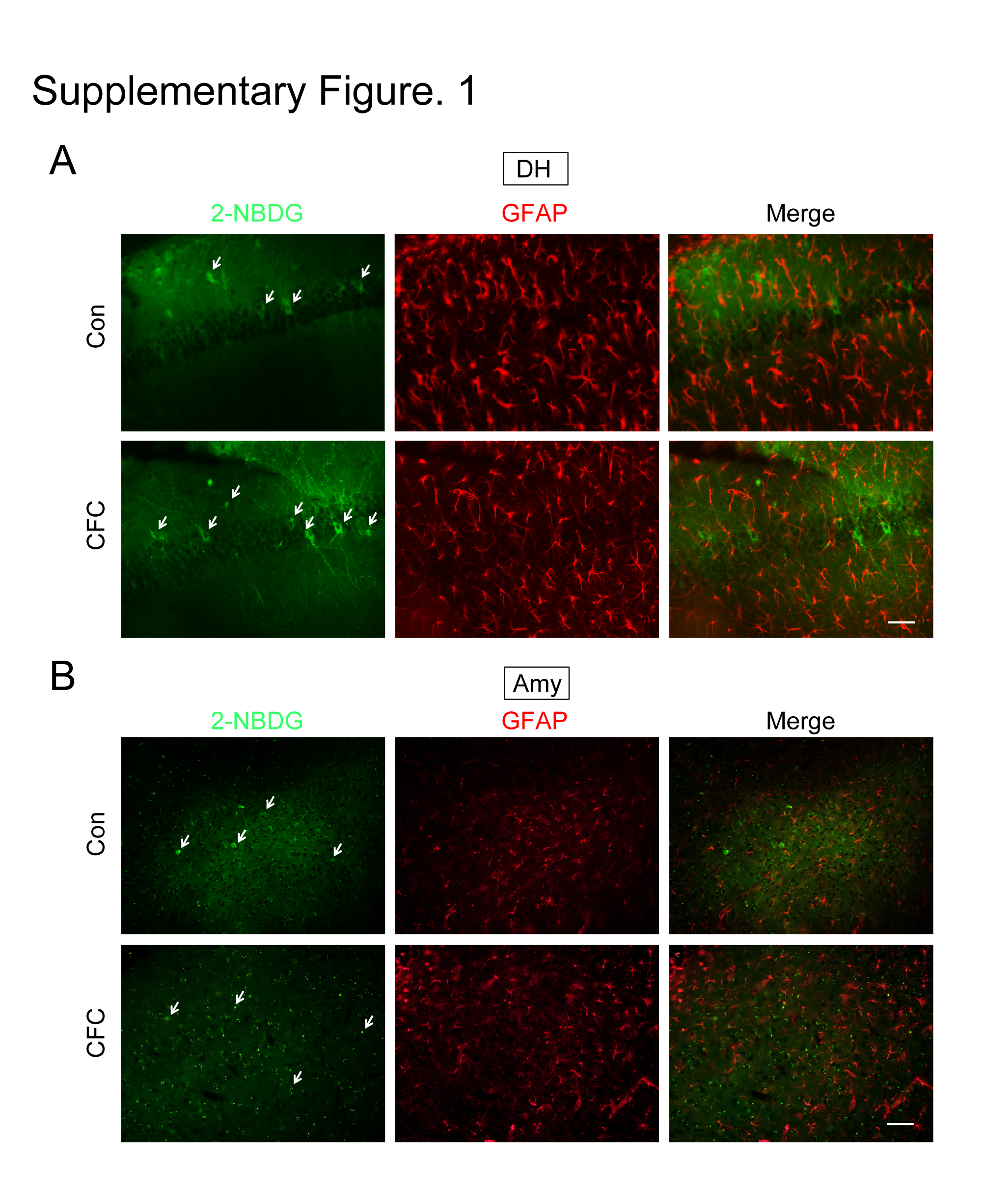

Supplement: FIGURE S1 — There is no overlap of 2-NBDG+ and GFAP+ positive cells under basal condition and CFC training in the DH and amygdala. (A) Representative fluorescence images of 2-NBDG and GFAP after microinjection of 2-NBDG into the DH (n = 4 per group). (B) Representative fluorescence images of 2-NBDG and GFAP after microinjection of 2-NBDG into the amygdala (n = 4 per group). White arrows represent only 2-NBDG+ cells. Scale bar = 50 μm. [file Image_1.tif]

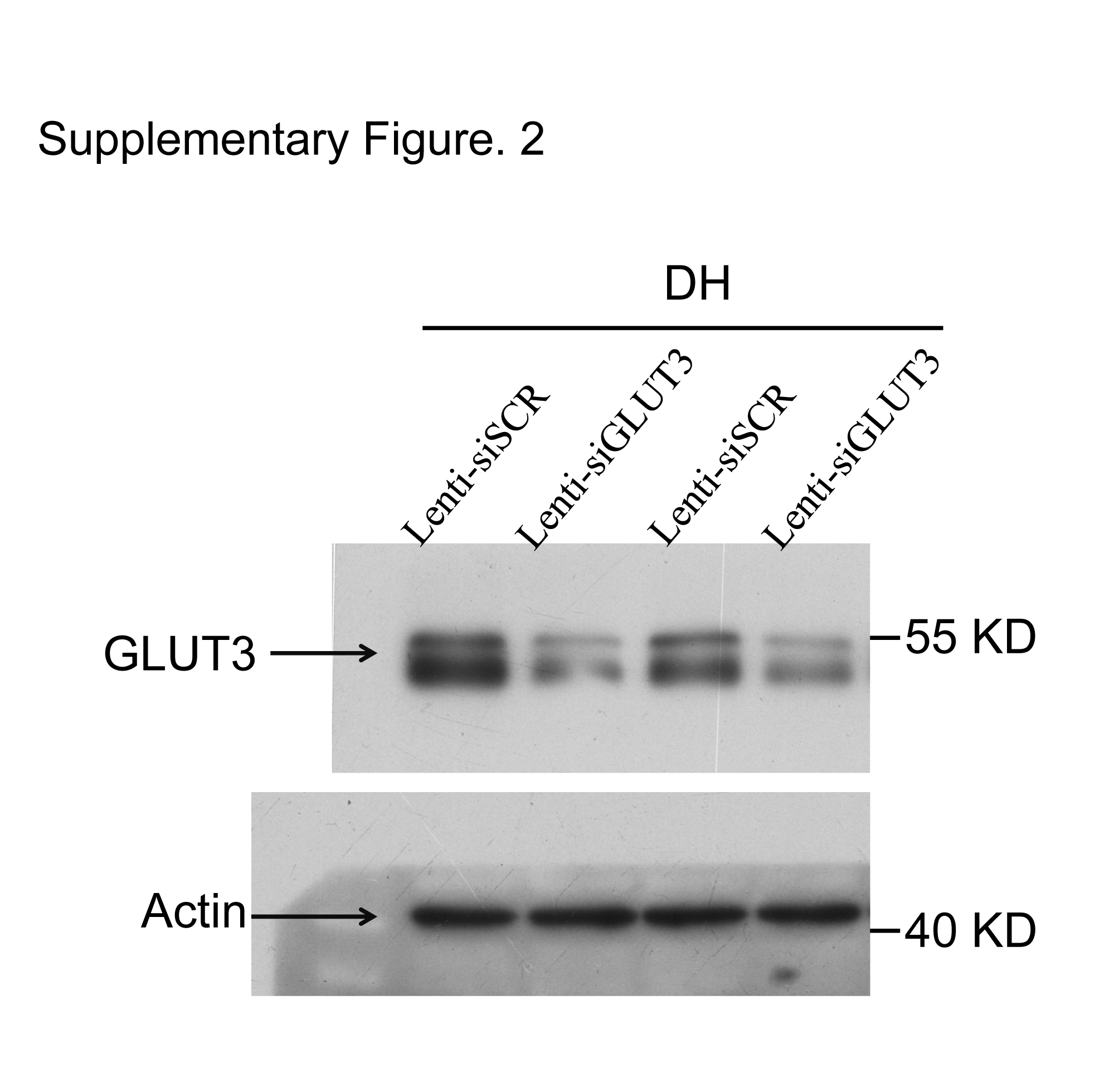

Supplement: FIGURE S2 — The original western blot images of Figure 5B. [file Image_2.tif]
